# The Certainty of Uncertainty: Potential Sources of Bias and Imprecision in Disease Ecology Studies

**DOI:** 10.3389/fvets.2018.00090

**Published:** 2018-05-22

**Authors:** Shelly Lachish, Kris A. Murray

**Affiliations:** ^1^Department of Zoology, University of Oxford, Oxford, United Kingdom; ^2^Department of Infectious Disease Epidemiology and Grantham Institute – Climate Change and the Environment, Imperial College London, London, United Kingdom

**Keywords:** imperfect detection, state misclassification, sensitivity, specificity, wildlife disease, host-pathogen, prevalence, disease impacts

## Abstract

Wildlife diseases have important implications for wildlife and human health, the preservation of biodiversity and the resilience of ecosystems. However, understanding disease dynamics and the impacts of pathogens in wild populations is challenging because these complex systems can rarely, if ever, be observed without error. Uncertainty in disease ecology studies is commonly defined in terms of either heterogeneity in detectability (due to variation in the probability of encountering, capturing, or detecting individuals in their natural habitat) or uncertainty in disease state assignment (due to misclassification errors or incomplete information). In reality, however, uncertainty in disease ecology studies extends beyond these components of observation error and can arise from multiple varied processes, each of which can lead to bias and a lack of precision in parameter estimates. Here, we present an inventory of the sources of potential uncertainty in studies that attempt to quantify disease-relevant parameters from wild populations (e.g., prevalence, incidence, transmission rates, force of infection, risk of infection, persistence times, and disease-induced impacts). We show that uncertainty can arise via processes pertaining to aspects of the disease system, the study design, the methods used to study the system, and the state of knowledge of the system, and that uncertainties generated via one process can propagate through to others because of interactions between the numerous biological, methodological and environmental factors at play. We show that many of these sources of uncertainty may not be immediately apparent to researchers (for example, unidentified crypticity among vectors, hosts or pathogens, a mismatch between the temporal scale of sampling and disease dynamics, demographic or social misclassification), and thus have received comparatively little consideration in the literature to date. Finally, we discuss the type of bias or imprecision introduced by these varied sources of uncertainty and briefly present appropriate sampling and analytical methods to account for, or minimise, their influence on estimates of disease-relevant parameters. This review should assist researchers and practitioners to navigate the pitfalls of uncertainty in wildlife disease ecology studies.

## Introduction

Wildlife disease ecology is a burgeoning field of research with important implications for wildlife and human health, the preservation of biodiversity, and the resilience of ecosystems. Monitoring pathogens in wild populations is undertaken for a variety of reasons (see Table 1 for examples), all of which require accurate assessments of pathogen occurrence or its derivatives to ensure that models, theory, and management recommendations are robust. Detecting and quantifying pathogen presence and prevalence in wild populations is challenging, however, because it is rarely possible to observe these complex systems without error, leading to biased and imprecise measurements (see Glossary). This uncertainty can arise at multiple levels of the sampling or diagnostic processes employed, from the choice of which sites and individuals to survey, to the processing of tissue samples in the lab (4, 11–13). Previous studies have recognised this multilevel nature of uncertainty, and in many cases methodological and statistical frameworks capable of accounting for hierarchical levels of observation error have been developed [e.g., by repeated sampling at each level to parse out non-detection biases from true absences, see discussion below; (11, 14, 15)]. Nevertheless, a mechanistic overview of how uncertainty arises in disease ecology studies remains lacking.

**Table 1:** Summary table listing examples of the varied objectives of disease ecology studies, along with key examples of each.

**Objectives of disease ecology studies**		**Examples**	**Key references**
1.	To quantify disease impacts in		
	1. Species of conservation concern	Tasmanian devils affected by Facial Tumour Disease	(1)
		Amphibians affected by chytridiomycosis	(2)
	2. Populations destined for translocation	European bison	(3)
2.	To map infection patterns		
	1. To help manage disease risks in endangered species or agricultural species	*Batrachochytrium dendrobatidis* in amphibians	(4)
		Tuberculosis in badgers	(5)
	2. To track disease spread	White nose syndrome in bats	(6)
3.	To understand host-pathogen dynamics and co-evolution		
	1. Under different ecological or environmental conditions	Mycoplasma in house finches	(7)
	2. Under varying degrees of anthropogenic influence	*Echinococcus multilocularis* in urbanised foxes	(8)
4.	To identify potential pathogens in animal hosts or vectors not yet circulating in human populations	Viral discovery efforts in wildlife	(9)
5.	To diagnose causes of unexplained illness or mortality events	Peste des petits ruminants in Saiga antelope	(10)

Sources of uncertainty have previously been attributed either to heterogeneity in the detectability of hosts, vectors, or their pathogens (imperfect detection) or to errors in disease state assignment [disease-state misclassification; (11, 16)]. In reality, however, uncertainty due to imperfect detection and state misclassification can arise in a variety of ways in studies of disease in natural populations, while other sources of uncertainty (e.g., incomplete taxonomic knowledge, demographic misclassification; see below) do not readily fit into this traditional dichotomy. This highlights the need to consider the sources, processes involved and implications of uncertainty more broadly.

Here we present an inventory of sources of uncertainty that could potentially occur when attempting to quantify disease-relevant parameters in wild populations (prevalence, incidence, transmission rates, force of infection, risk of infection, persistence times, and disease-induced impacts; see Glossary). Many of these (for example, unidentified crypticity among vectors, hosts or pathogens, a mismatch between the temporal scale of sampling and disease dynamics, demographic or social misclassification: see discussion below) may not be immediately apparent, even to seasoned investigators, or even identifiable without preliminary study. As such, the bias and lack of precision introduced by them cannot always be adequately accounted for by post-hoc statistical adjustment (11, 17). In such instances, detailed understanding of the host-pathogen system and the development of more nuanced methodological approaches may be required.

## Sources of Uncertainty in Disease Ecology Studies

Based on a structured literature review (Appendix 1, Data Sheet S1), we classified sources of uncertainty into six broad (and potentially overlapping) categories. We found uncertainty could arise due to: (1) intrinsic biological factors associated with the interaction between hosts and pathogens; (2) demographic or state misclassification; (3) incomplete taxonomic knowledge of host-pathogen systems; (4) a mismatch of sampling scales; (5) imprecision of diagnostic methods; and (6) extrinsic environmental factors that may have additional modifying effects within each of the other categories. Details of these sources of uncertainty, the processes involved and an indication of the potential bias and imprecision in disease-relevant parameters are presented in Table 2.

### A. Intrinsic Biological Factors

#### A.i. Variation in Detectability

**Figure 1:**
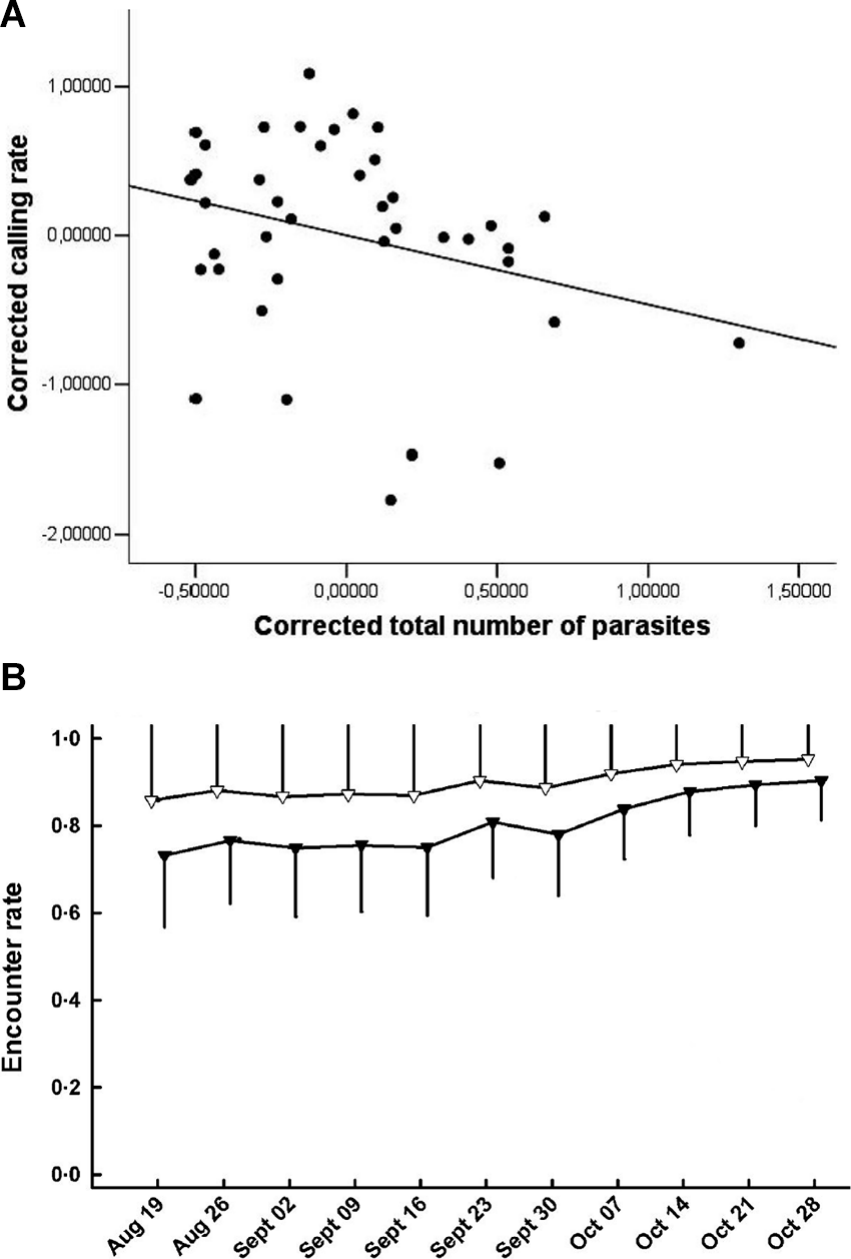
**(A)** Calling rate of treefrogs (*Hypsiboas prasinus*) versus total number of helminth parasites. Corrected calling rate and total number of parasites are the residuals of a regression of body mass; the line is illustrative (*Adapted from Madelaire et al. (23), with permission from the Journal of Herpetology*); **(B)** Encounter rate of house finches that were infected with *Mycoplasma gallisepticum* (black triangles) or not infected (white triangles) *[Adapted from Faustino et al. (7), with the permission of John Wiley and Sons*].

It is widely recognized that detection of organisms in their natural environment is rarely perfect and that detectability (encounter, capture or sighting rate) can vary as a function of time and numerous biotic and abiotic factors (18). One of these factors can be infection status (Table 2,A.i.). Differential detectability between infected and uninfected individuals (hosts or vectors) can occur either because the pathogen directly manipulates individual behaviour or because the behaviour of individuals changes due to deterioration in their physiological condition, which might further scale with infection intensity (16, 19–22). These processes can modify the movement patterns or conspicuousness of individuals, changing their trappability, visibility, or propensity to migrate out of the study site. For example, Brazilian treefrogs (*Hypsiboas prasinus*) with more intense helminth infections exhibit reduced mating call frequencies, which could make them less detectable during surveys (23; Figure 1A). Similarly, house finches infected with *Mycoplasma gallisepticum* infection suffer impaired vision and display reduced activity levels, resulting in lower recapture rates for infected compared to uninfected individuals (Figure 1B; 7, 24). When detectability is “imperfect” and differs as a function of infection status or intensity then unadjusted estimates of disease-relevant parameters may be biased (16, 20).

A further implication of differential detectability of infected vs uninfected individuals is that observed temporal patterns in disease dynamics may simply be spurious artefacts of temporal variation in the probability of detecting infected vs uninfected individuals (20, 25, 26). Using simulations, Jenelle et al. (20) demonstrate how temporal variation in detection probabilities of infected individuals could suggest a cyclic pattern of disease prevalence, even when the true prevalence is constant over time. Equally, real seasonality in disease dynamics (see below and Table 2,F) may be masked by contrasting temporal patterns of detection of hosts, vectors, or pathogens (20).

#### A.ii. Variation in Distribution and Intensity of Pathogens Among Hosts

**Figure 2:**
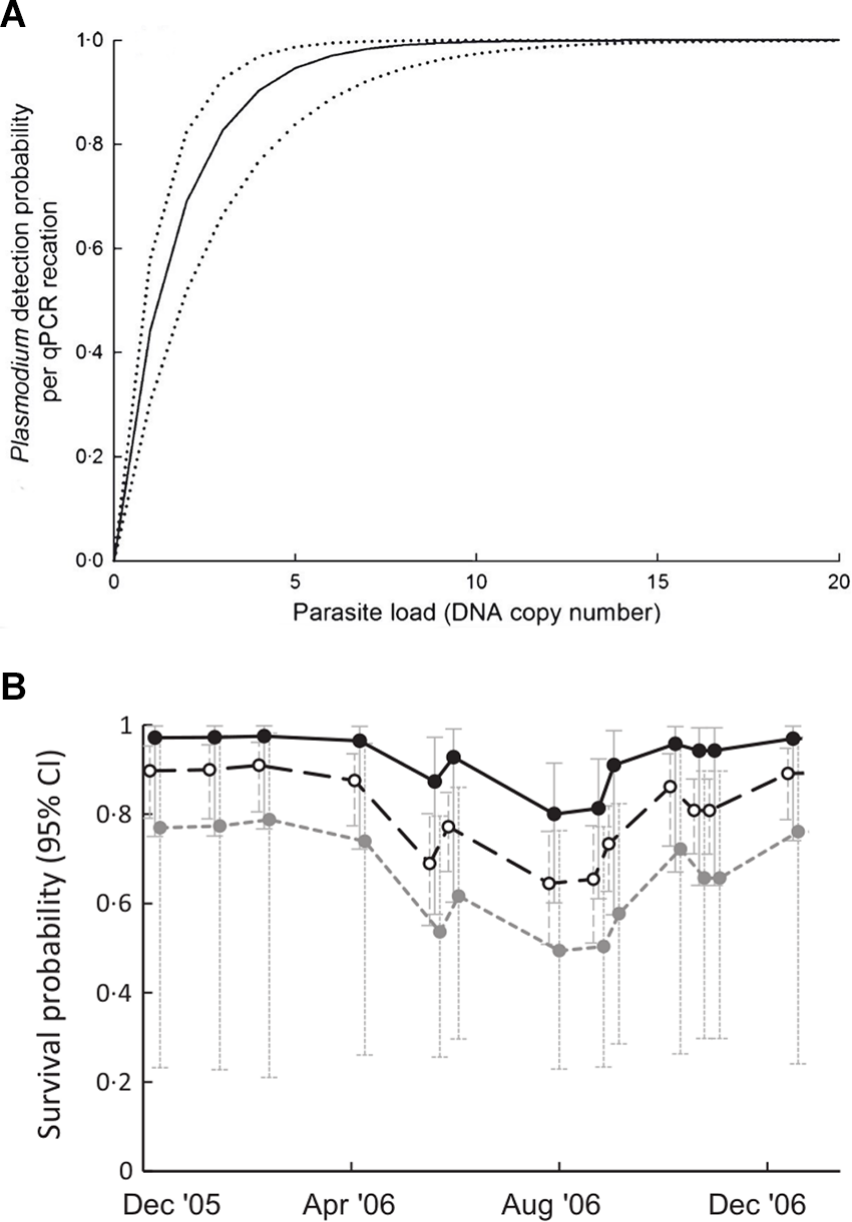
**(A)** Relationship between parasite load (DNA copy number) and the probability of detecting *Plasmodium* infection by qPCR in blue tits (*Cyanistes caeruleus*). Dotted lines are 95% confidence intervals (*Adapted from (12), with the permission of John Wiley and Sons*); **(B)** relationship between infection with Bd and survival probability of male *Litoria rheocola* as a function of pathogen load: uninfected (○), 1–4 zoospores (●) and >4 zoospores (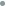) *[Adapted from (29), with the permission of John Wiley and Sons*].

Pathogen infections in natural populations are often characterised by aggregated or over-dispersed distributions among individuals within populations (27; Table 2,A.ii.). That is, most infected individuals harbour low parasite burdens, while relatively few harbour high parasite burdens. Although typical of macroparasite infections (e.g., helminths), microparasites can also exhibit over-dispersed distributions in terms of variable patterns of infection intensity among hosts (12, 28, 29). For example, Grogan et al (29) showed that the distribution of *Batrachochytrium dendrobatidis* (Bd) load between amphibian hosts is highly over-dispersed. Despite its long-recognised importance for disease transmission rates and host-pathogen population dynamics (30), the effect of pathogen aggregation and variation in disease intensity on the detection and estimation of disease occurrence and impacts has only recently been established. For example, Shin et al. (31) showed that detection of Bd infection is unreliable in individuals with low Bd loads. Similarly, the probability of detecting *Plasmodium* infections in avian blood increases with pathogen load (12; Figure 2A). Thus, pathogen aggregation can generate bias in disease-relevant parameters via increasing the likelihood of errors in disease state assignment (i.e., state misclassification) and will be particularly relevant when the sensitivity of diagnostic tests is low (see discussion below and Table 2E). Pathogen aggregation can also generate imprecision and bias in estimates of the magnitude of disease impacts on individuals when the magnitude of disease-induced impacts varies with parasite burden or the intensity of infection (12, 29, 31, 32). For example, Grogan et al. (29) demonstrated that survival of the common mist frog (*Litoria rheocola*) was related to the burden of infection with Bd, and that accurate knowledge on infection dynamics in their system necessitated accounting for pathogen overdispersion (Figure 2B).

### B. Demographic or Social Misclassification

**Figure 3:**
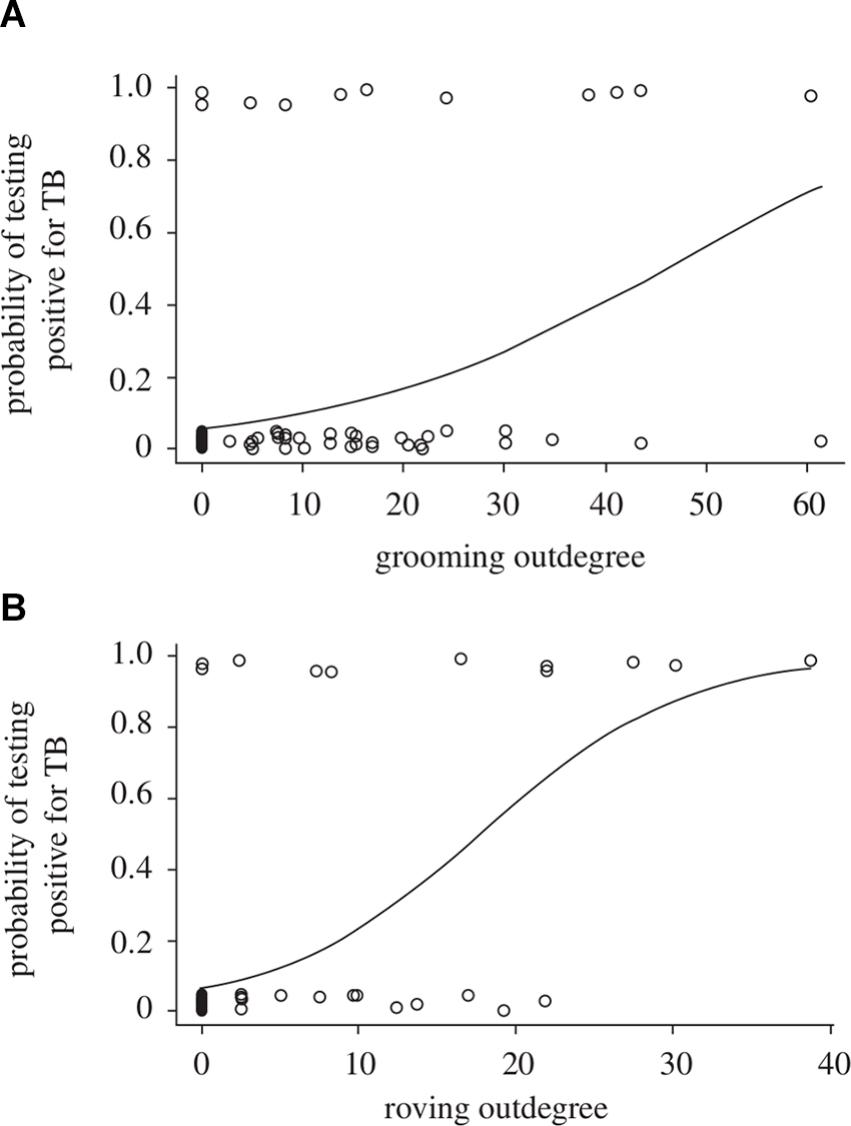
Probability of individual meerkats testing positive for tuberculosis as a function of **(A)** the extent to which they groom others (grooming outdegree) and **(B)** the extent of intergroup excursions by males (roving male outdegree). *[Adapted from (36), with the permission of The Royal Society*].

Just as there can be uncertainty in the assignment of disease-state for individuals in natural populations, there can also be uncertainty in assigning individuals to demographic or social classes (i.e., age, sex, social status: Table 2B). For example, imperfect methods of ageing individuals will produce biased and imprecise estimates of age prevalence curves or of demographic impacts (33). An inability to accurately sex individuals (e.g., in juveniles or where sexual dimorphism is absent) or to accurately infer social groups or social hierarchies could introduce further biases, as sex differences and social structure can strongly influence disease transmission and dynamics (34). For example, in badger (*Meles meles*) populations individuals that are more socially isolated from their group are at greater risk of tuberculosis (Tb) infection (35), while in meerkat (*Suricata suricatta*) populations highly connected group members (those that groom more) and roving males are more likely to be infected with Tb (36; Figure 3A,B). The effect of social status on infection risk can also differ between demographic classes, as was recently shown for spotted hyenas (*Crocuta crocuta*) infected with canine distemper: high social rank increased infection risk for adults and subadults (as they had higher contact rates and disease exposure) but decreased infection risk for cubs (as they were in better physiological and immunological condition; (37). Furthermore, if infection is demographically biased (i.e., when infection varies with age, sex, or social status) then encounter rates could also vary between those classes, resulting in additional uncertainty in estimates of age prevalence curves, demographic impacts, or disease dynamics (38).

**Table 2:** Summary of key sources and processes that can generate bias or lack of precision in estimates of disease-relevant parameters obtained in disease ecology studies.

**Potential sources of uncertainty**	**Process**	**Potential bias and imprecision in disease-relevant parameters**
**A. Intrinsic biological factors**		
i. Variation in detectability	Detectability of uninfected vs infected individuals may differ because the pathogen directly manipulates or impacts host behaviour or physiological condition.	If infected individuals are detected less frequently than uninfected individuals, estimates of prevalence and transmission will be underestimated, while estimates of disease impacts and recovery rates will be overestimated (and *vice-versa*). The direction and magnitude of bias and imprecision will depend on the extent of heterogeneity and temporal variation in detection rates.
ii. Variation in distribution and intensity of pathogens among hosts	Pathogen load and disease severity often exhibit aggregated distributions among hosts, which may result in misclassification of disease state in individuals with minor symptoms or low parasite burdens.	If individuals are misclassified as uninfected, estimates of prevalence and transmission will be underestimated and estimates of recovery rates overestimated.
**B. Demographic or social misclassification**	Error in assigning individuals to demographic or social classes (e.g., sex, age, social status).	Direction and magnitude of bias and degree of imprecision in estimates will depend on the direction and extent of assignment errors.
**C. Incomplete taxonomic knowledge**		
i. Taxonomic crypticity	Multiple, cryptic host, vector or parasite species are present but may be overlooked due to lack of taxonomic resolution.	Direction and magnitude of bias and imprecision will depend on the proportion of cryptic or rare species present, the rarity of the rare entities, the complexity of the multi-host-pathogen species assemblage and the degree of sampling effort that is feasible to estimate or detect the assemblage(s) being catalogued.
ii. Rare or less detectable species	Logistical constraints restrict sampling completeness and may preclude the detection of rarer or less detectable entities.	
iii. Multi-host or multi-pathogen systems	Coinfections or variation in abundance, diversity or susceptibility among hosts may alter infection dynamics	
**D. Mismatch of sampling scale and process scale**		
i.Temporal	Temporal scale of sampling does not match the temporal scale of disease dynamics, or sampling effort is disproportionate in time.	Missed infections will result in underestimates of survival of uninfected hosts, overestimates of survival of infected hosts, and underestimates of infection rates.
ii.Spatial	Spatial extent of sampling does not match spatial scale of disease dynamics, or sampling effort is disproportionate in space.	Direction and magnitude of bias and imprecision will depend on the study system and the sampling regime adopted. Sampling biases (e.g., along roads) may inflate estimates of probability of occurrence.
**E. Diagnostic Procedures**		
i. Imperfect sensitivity or specificity of the diagnostic assay	Diagnostic tests may either fail to detect pathogens when present (false negative) or produce positive diagnoses in the absence of infection (false positive), or both.	The presence of false negatives (or false positives) in a sample will negatively (or positively) bias estimates of pathogen prevalence, with errors propagating to other parameter estimates. Magnitude and direction of bias and imprecision will depend on the sensitivity and specificity of the diagnostic assay, degree of pathogen aggregation among hosts, threshold titre values chosen, and potential for cross-reactivity in serology studies.
ii. Variability between entities making the diagnosis	Sensitivity or specificity of a diagnostic assay can vary between laboratories, technicians or observers as a function of procedures, equipment, or expertise.	
iii. Tissue type sampled	Infection presence or detectability may vary by tissue type.	
**F. Extrinsic environmental factors**	The proximal and distal effects of extrinsic environmental factors may influence a range of components of host-pathogen systems, many of which are described above, and can be considered a cross-cutting source of potential bias/uncertainty.	Overlooking potential effects of environmental factors on disease dynamics may produce biased and imprecise parameter estimates, poorly characterised disease dynamics, or erroneous inferences on the mechanisms driving them. Magnitude and direction of bias and imprecision will be highly variable and dependent on the specific study system.

### C. Incomplete Taxonomic Knowledge

#### C.i. Taxonomic Crypticity

**Figure 4:**
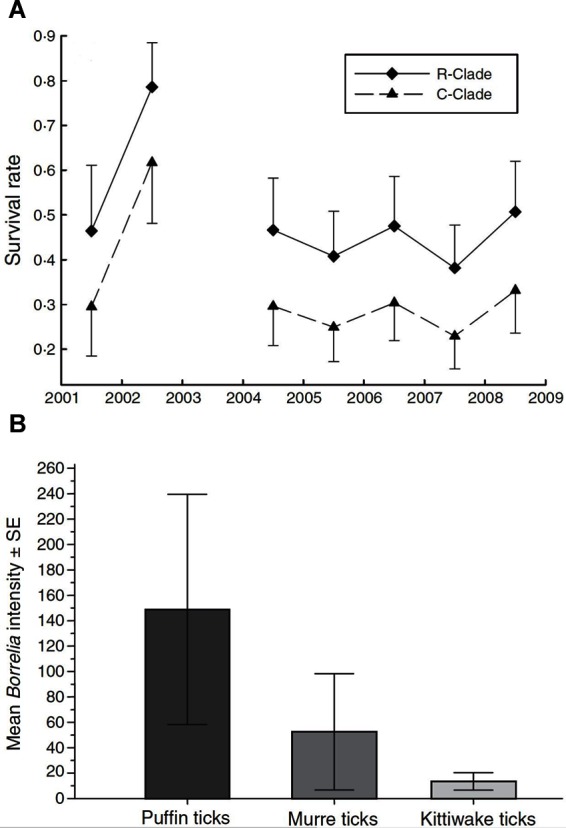
**(A)** Survival rates (±95% CI) of blue tits infected with two strains of avian malaria (*Plasmodium relictum *◆ R-clade; *P. circumflexum* ▲ C-clade). (Adapted from Lachish et al. (21), *with the permission of John Wiley and Sons*); **(B)** Intensity of infection of Lyme borreliosis bacteria (mean spirochetes ± SE) in three morphologically cryptic avian tick races associated with puffins, murres, and kittiwakes. *[Adapted from (39), with the permission of John Wiley and Sons*].

Erroneous or biased inferences on disease dynamics may arise when multiple, phenotypically indistinguishable but genetically distinct host, vector or parasite species are present but are cryptic, and therefore, overlooked (21, 39), (Table 2,C.i.). Virulence can vary among parasite species (40). Different parasite species or morphotypes can differ in the nature of their impacts on hosts (21), or in their detectability within hosts (41). For example, compared to uninfected individuals, blue tits (*Cyanistes caeruleus*) infected with *Plasmodium circumflexum* experience lower survival, while those infected with *Plasmodium relictum* experience lower reproductive success (21; Figure 4A). These contrasting impacts on blue tit fitness were obscured when the identity of the two cryptic malaria species was ignored (21). Cryptic vector species can vary in their contribution to local infection dynamics, while the detectability of parasites in vectors can also vary among vector species (39). For example, Gomez et al., (39) showed that infection intensity with *Borrelia spp* varied strongly among cryptic tick races (Figure 4B), leading to variable detection rates and vector-specific biases of between 4 and 30% when raw counts were used to calculate prevalence.

Crypticity among host species can also influence disease processes and may bias estimates of the distributions of hosts or disease, or provide previously unidentified explanations for observed disease distributions (42). For example, spatial heterogeneity in Lassa fever outbreaks in humans was resolved only when the cryptic phylogeography of its reservoir host species, the rodent *Mastomys natalensis*, was recognised (42). These examples show that crypticity can generate uncertainty in disease-relevant parameters via “imperfect detection” (e.g., when detection rates vary among cryptic morphotypes (21, 39), via “state misclassification” (e.g., when diagnosis varies with virulence or severity which differ among the cryptic morphotypes (39), via both processes concurrently, or via other means (e.g., when morphotypes impact hosts differently; (21) or when incomplete knowledge limits the phylogeographic range of investigation; (42).

#### C.ii. Rare or Less Detectable Species

**Figure 5:**
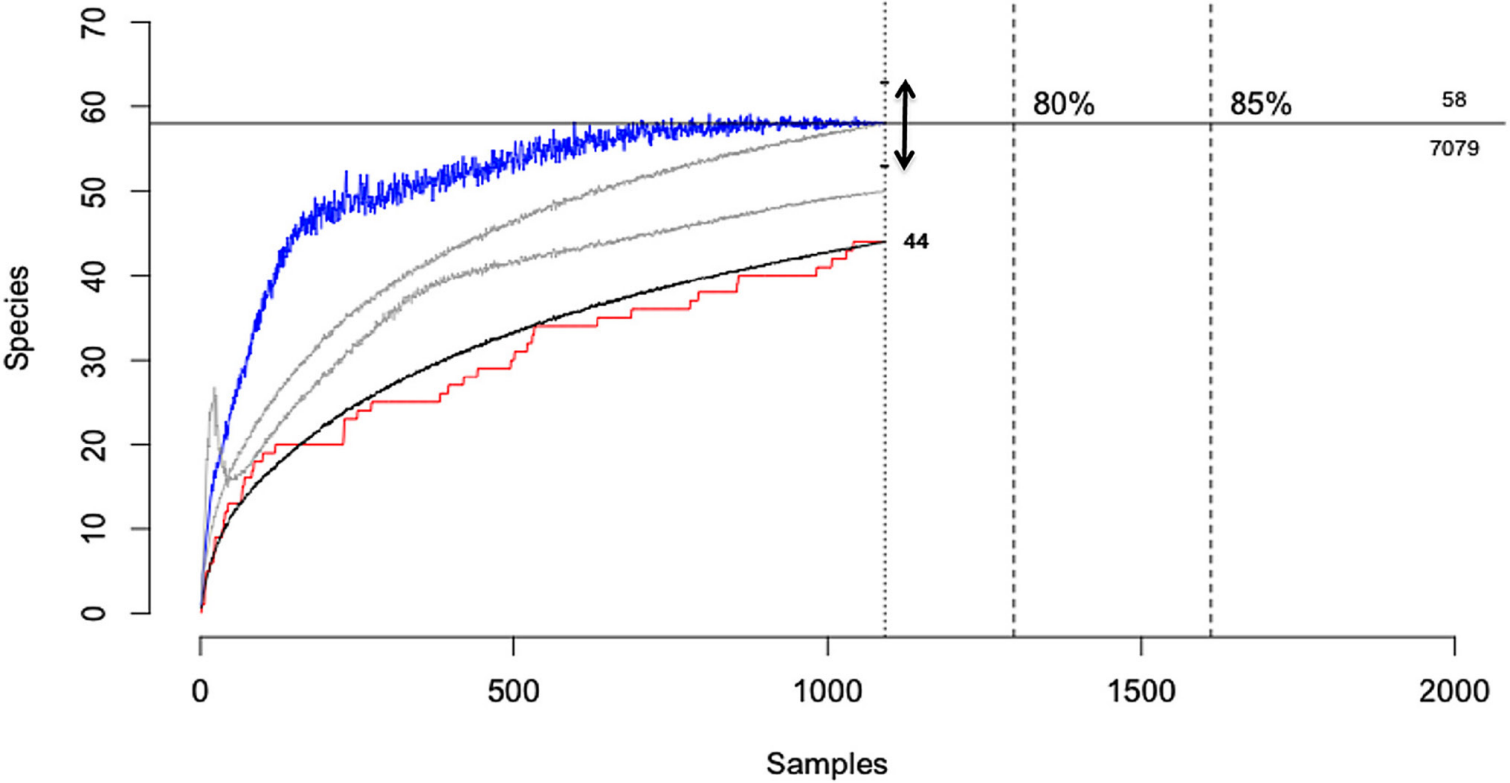
Viral discovery curves for pathogens of the Indian Flying Fox (*Pteropus giganteus*) using PCR estimated from observed detections using three statistical models. The horizontal line shows the total estimated diversity (58 viruses) corrected for detectability and the effort required to discover 100% of the estimated diversity (7,079 samples). Black line, the rarefaction curve; red line, accumulation of novel viruses over samples tested; blue line, Chao2 estimator with arrow = 95% CI; gray lines, ICE and Jackknife estimators; *[Adapted from (9), *under creative commons licence].

Another form of uncertainty may emerge from the incomplete characterisation of biological assemblages due to the non-detection of rare or low detectability species (Table 2,C.ii.), which constitutes an extreme form of “imperfect detection”. For example, when characterising the diversity of micro-organisms within a host (e.g., the microbiome) or when undertaking pathogen discovery campaigns in wildlife hosts, there will typically be diminishing returns in terms of new species detections with increasing sampling effort. This arises because more common or easily detectable species in an assemblage are catalogued early while rarer or less detectable species require greater effort (9, 43). For both hosts and pathogens, sampling campaigns will rarely census entire communities due to logistical constraints and as such there will be uncertainty when estimating host breadth, pathogen species richness or other diversity metrics. For example, when characterising the viral diversity of the wild megabat *Pteropus giganteus*, Anthony et al., (9), detected 44 viruses from 1092 samples. However, methods to account for imperfect detection suggested that a further 14 viruses remained undetected in this host, with the amount of testing required to detect them all estimated to be nearly seven-fold the number actually tested (9; Figure 5). This example illustrates how observations of host-pathogen systems can be directly biased by sampling completeness, which will always be constrained by logistical considerations.

#### C.iii. Multi-Host or Multi-Pathogen Systems

**Figure 6:**
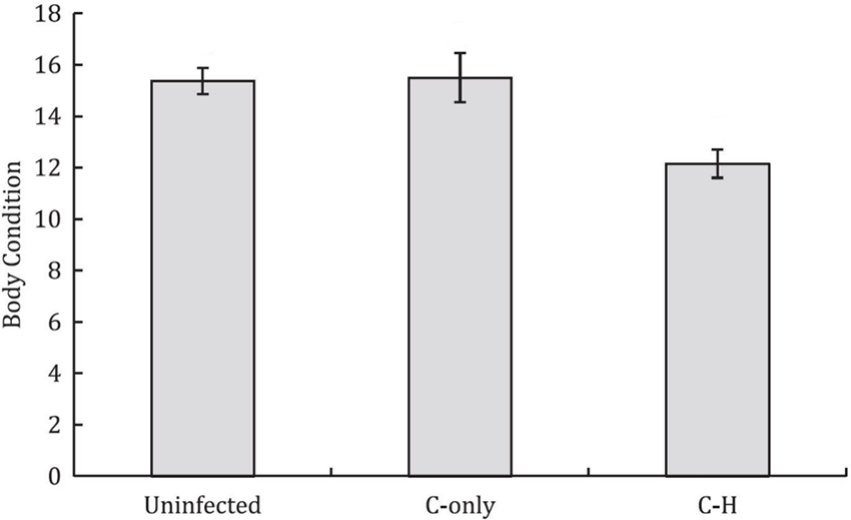
Buffalo coinfected with two gastrointestinal parasites (*Cooperia-Haemonchus*) exhibit lower body condition compared to uninfected and *Cooperia*-only singly-infected buffalo (means ± SE are shown). *[Adapted from (45), *under creative commons licence].

Alongside unacknowledged taxonomic crypticity, and the presence of rare species, potential uncertainty in disease-relevant parameters can arise when multi-pathogen or multi-host dynamics are present but ignored (Table 2,C.iii). Coinfections can involve both antagonistic and synergistic interactions between pathogens within hosts, which can alter the outcome of infection (positively or negatively) and thus influence disease dynamics and host fitness (44–46). For example, Budischak et al., (45) showed that body condition was lower in buffalo coinfected with two gastrointestinal macroparasite species (*Cooperia* and *Haemonchus*) compared to uninfected or singly-infected individuals (Figure 6). Likewise, for pathogens capable of infecting multiple host species, the diversity and abundance of alternative reservoir hosts and their relative susceptibility or competence, can alter the impact of disease on the focal hosts and disease dynamics within the host’s population (28, 47–49). For example, Kilpatrick et al., (47) showed that variability in host competence and mosquito feeding patterns results in extreme heterogeneity in the transmission of West Nile virus among communities of avian hosts. Such processes broadly related to the epidemiological complexity of a disease can have follow-on effects that may also introduce other forms of bias and uncertainty; for example, mapping efforts for increasingly complex human diseases, as crudely measured by the presence and number of transmission sources (e.g., environmental, reservoir hosts, vectors, human-human) tend to be of lower quality than for simpler diseases (50). Although both multi-pathogen coinfections and multi-host pathogens are common, their effects and dynamics in wild host populations in natural settings remain poorly described.

### D. Mismatch of Sampling Scale and Process Scale

#### D.i. Temporal Scales

Uncertainty in estimates of disease-relevant parameters can arise when the temporal or spatial sampling scales do not match those at which the disease dynamics operate (Table 2,D). For example, in many disease ecology studies the frequency of data collection occurs on a longer time scale than the disease dynamics (1, 2, 51). If hosts are only monitored seasonally or annually but the progression from infection to death, or from infection to recovery, occurs over weeks or months, then individuals can acquire infection and die, or acquire and lose infections, without these events appearing in the data (16, 17, 21).

#### D.ii. Spatial Scales

Inferences regarding disease dynamics may also vary as a function of the spatial extent of sampling relative to the area that determines pathogen dynamics (Table 2,D.ii. ; 52, 53). For example, the relationship between biodiversity and infection risk often depends strongly on the spatial scale of sampling (53). Studies of tick-borne Lyme disease conducted at small (within-forest) spatial scales reveal positive associations between disease risk (to humans) and host biodiversity (the so-called “amplification effect”), whereas those conducted at larger scales reveal the opposite (i.e., a “dilution effect”; (54). Similar conflicting inferences regarding the relationship between host biodiversity and the risk of infection with West Nile Virus have been demonstrated in studies conducted at small (48) and large spatial scales (55). A mismatch of spatial sampling scale is most likely to occur for zoonotic or vector-borne pathogens or those with complex life cycles, because the production of infective-stages may be decoupled spatially from the dynamics of the infection within the target host species (48, 54–56). The uncertainty generated by a mismatch of sampling scales cannot readily be classified as either ‘imperfect detection’ or “state misclassification”.

Disproportionate sampling effort in both space and time can be considered further examples of potential mismatches in scale that can introduce uncertainty in disease ecology studies (Table 2,D). For example, many datasets on disease occurrence are compiled from non-random “convenience” samples of individuals or locations, such as from passive surveillance of sick and dead wildlife. Thus, the sample units are not selected according to defined rules from the pool of all possible sample units that conceptually represent the population of interest (52). This can preclude calculating true probabilities of occurrence from a sample, resulting in biased and imprecise estimates of disease prevalence or other population parameters (52). This issue extends to the use of species distribution models (SDMs; also known as ecological niche models) developed from “presence-only” occurrence datasets. This approach is increasingly being applied in disease studies to map infection risk since robust records of disease absence are usually unavailable (e.g., in analysis of museum specimens) or cannot be accurately verified (due to sampling limitations; (57). In these cases, the distribution of reported occurrences are often tightly correlated with the distribution of reporting or observation effort, potentially resulting in misleading representations of disease distributions in model outputs (58, 59).

### E. Diagnostic Procedures

#### E.i. Imperfect Sensitivity or Specificity of the Diagnostic Assay

It is well recognised that most pathogen detection methods are imperfect, resulting in errors in disease state assignment (Table 2,E). This is true both for diagnoses made via laboratory analyses of field-collected tissue samples and those made via observational assessments of host symptoms in the field (11, 12, 15). Diagnostic tests with <100% sensitivity will produce negative diagnoses when a pathogen is present but not detected (false negatives), while tests with <100% specificity will produce positive diagnoses in the absence of infection (false positives; Table 2E.i). Less well recognised is how these properties interact with other potential sources of uncertainty. For example, the accuracy of diagnostic tests can vary with the intensity of infection in hosts, meaning they can be inconsistent when pathogen distributions are aggregated [see section above and (12, 15, 29)]. Quantitative PCR-based assays can fail to detect infections with low DNA copy number (low parasite load), as has been demonstrated for the detection of Bd (15) and avian malaria (12; Figure 2A). Meanwhile, observational diagnoses may fail to detect asymptomatic individuals or those with minor symptoms (1, 7). Accounting for uncertainty due to diagnostic test accuracy will be especially necessary in studies of pathogens with over-dispersed distributions among hosts (29).

Issues of low sensitivity and specificity are particularly problematic in studies that use serological data to infer infection status because state assignment is based on arbitrary threshold values, which can increase the likelihood of false negatives and consequently bias estimates of disease-relevant parameters (60). In addition, cross-reactivity in serology can occur in the presence of unidentified pathogen diversity, increasing the likelihood of false positives (60, 61). Indeed, the presence of cryptic pathogen species (as discussed above) could lower the specificity of many diagnostic tests (60). There can be additional uncertainty in inferences obtained from studies that use serology-derived measures of disease when knowledge of the serological outcomes following infection is lacking (e.g., the probability that an infected individual will seroconvert; how pathogen dose and route of inoculation affect the induction of a host antibody response; the duration of the antibody response to infection; and relationship between antibody status and resistance to pathogen infection; (61, 62).

#### E.ii. Variability Between Entities Making the Diagnosis

Another potentially common, but rarely considered, source of uncertainty in the diagnosis of infection is the variability in diagnostic accuracy that can exist between different laboratories, technicians or observers because of differences in expertise, equipment or procedures (63; Table 2,E.ii.). Such variation in diagnostic accuracy may lead to erroneous inference when comparing prevalence and disease dynamics across studies and regions (18, 41).

#### E.iii. Tissue Type Sampled

Finally, the choice of which tissues to sample within hosts can also induce uncertainty in parameter estimates because infection or the composition of pathogen assemblages may vary among tissue types (64; Table 2,E.iii). For example, avian plasmodium is less detectable in blood than in other tissues [via PCR; (65)]. As most studies of avian malaria infections in wild birds diagnose infection in blood, researchers must acknowledge the likelihood that parameter estimates are biased because of “missed” infections (65).

### F. Extrinsic Environmental Sources of Uncertainty

**Figure 7:**
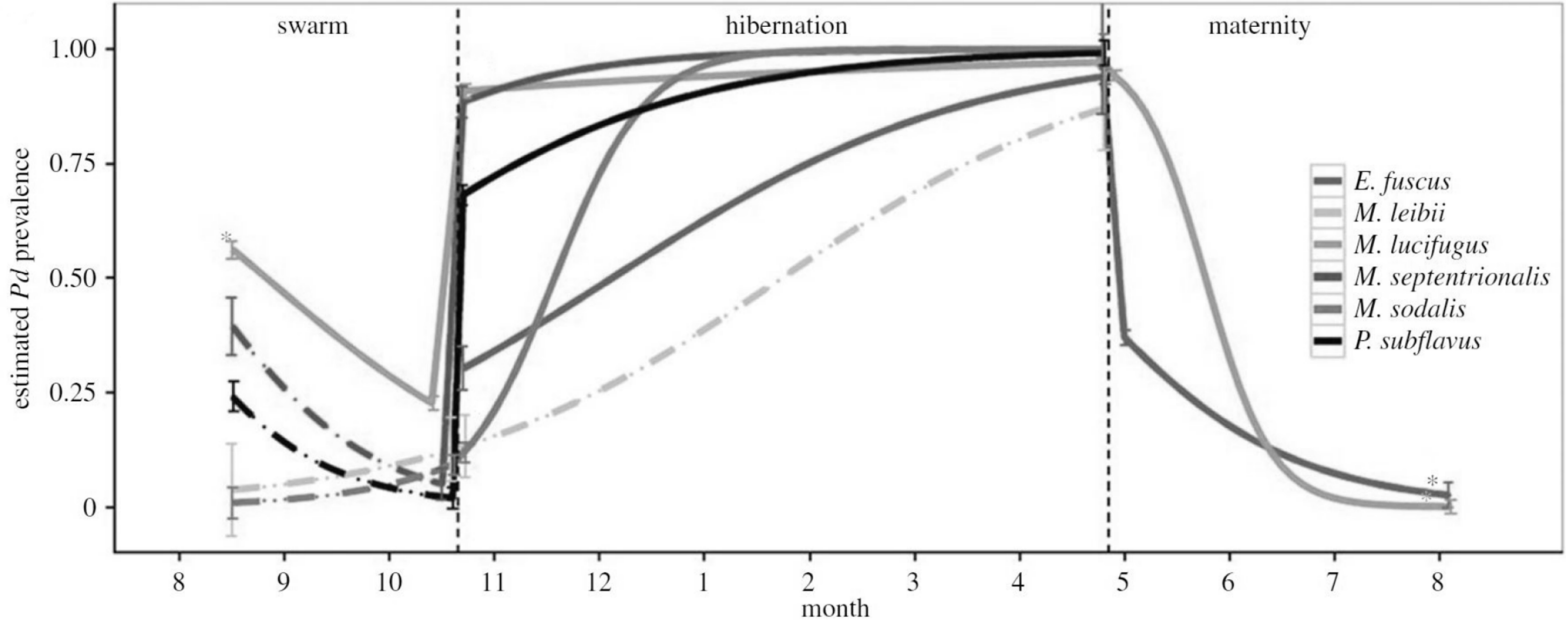
Model results showing that the peak predicted prevalence (mean ± SE) of white nose virus (*Pseudogymnoascus destructans*) for six species of bats coincides with onset of hibernation. *[Adapted from (12), with the permission of The Royal Society]*.

External factors related to the environment often have pervasive effects, both proximal and distal, on the components of host-pathogen systems, and as such, environmental covariates can be considered a crosscutting source of potential uncertainty in disease ecology studies (Table 2,F). For example, seasonal changes in the incidence of infectious diseases is common. Seasonal forcing of disease dynamics occurs for a variety of ecological reasons, including seasonal pulses of births and deaths, seasonal changes in host immunity or parasite vigour, or because of seasonal changes in host behaviours (e.g., hibernation, migration, mating) that result in temporal changes in host contact rates, or variation in encounters with or proliferation of infective stages in the environment (25, 66–69). For instance, seasonal transmission of the fungus *Pseudogymnoascus destructans,* the cause of bat white nose syndrome, is primarily driven by changes in host physiology related to hibernation, which facilitates fungal growth in North American caves during winter (70; Figure 7). Less regular or longer-term environmental influences can similarly affect disease dynamics: for instance, if outbreaks are related to unusual weather events (flood, drought) or transmission varies by longer term cycles or changes in climate (ENSO, climate change). For example, outbreaks of the coral disease, atramentous necrosis, are associated with increased rainfall, greater particulate runoff, and higher water temperatures, all of which are predicted to increase under future climate change (69). Disease ecology surveys that overlook the effects of seasonality and other environmental factors on disease transmission may thus produce biased and imprecise estimates of disease-relevant parameters, poorly characterised disease dynamics, or fail to identify important mechanisms driving them (20, 26, 52). At worst, such studies may entirely fail to detect pathogens; for example, by only sampling annually or in a temporally ad-hoc manner (see also mismatch in scale section above; (52).

Spatial variation in environmental covariates can similarly introduce uncertainty in wildlife disease studies. For example, host species may utilise different habitat types. This could lead to differences in host detectability during disease surveys (52) or could directly affect the host-pathogen relationship. For example, the dynamics of chytridiomycosis in amphibians vary according to microclimate, which itself varies by habitat type, resulting in fine scale “environmental refugia” from disease [e.g., open vs closed canopy tropical stream habitats, (71)]. Because the impact of disease is lower in refugia (due to limited fungal growth and/or enhanced frog immunity), ignoring the presence and variability of favourable or unfavourable habitats in a landscape can bias inferences and compromise efforts to map disease risk or plan conservation actions (e.g., translocation of critically endangered species; (71).

## Accounting for Uncertainty in Disease Ecology Studies

**Figure 8:**
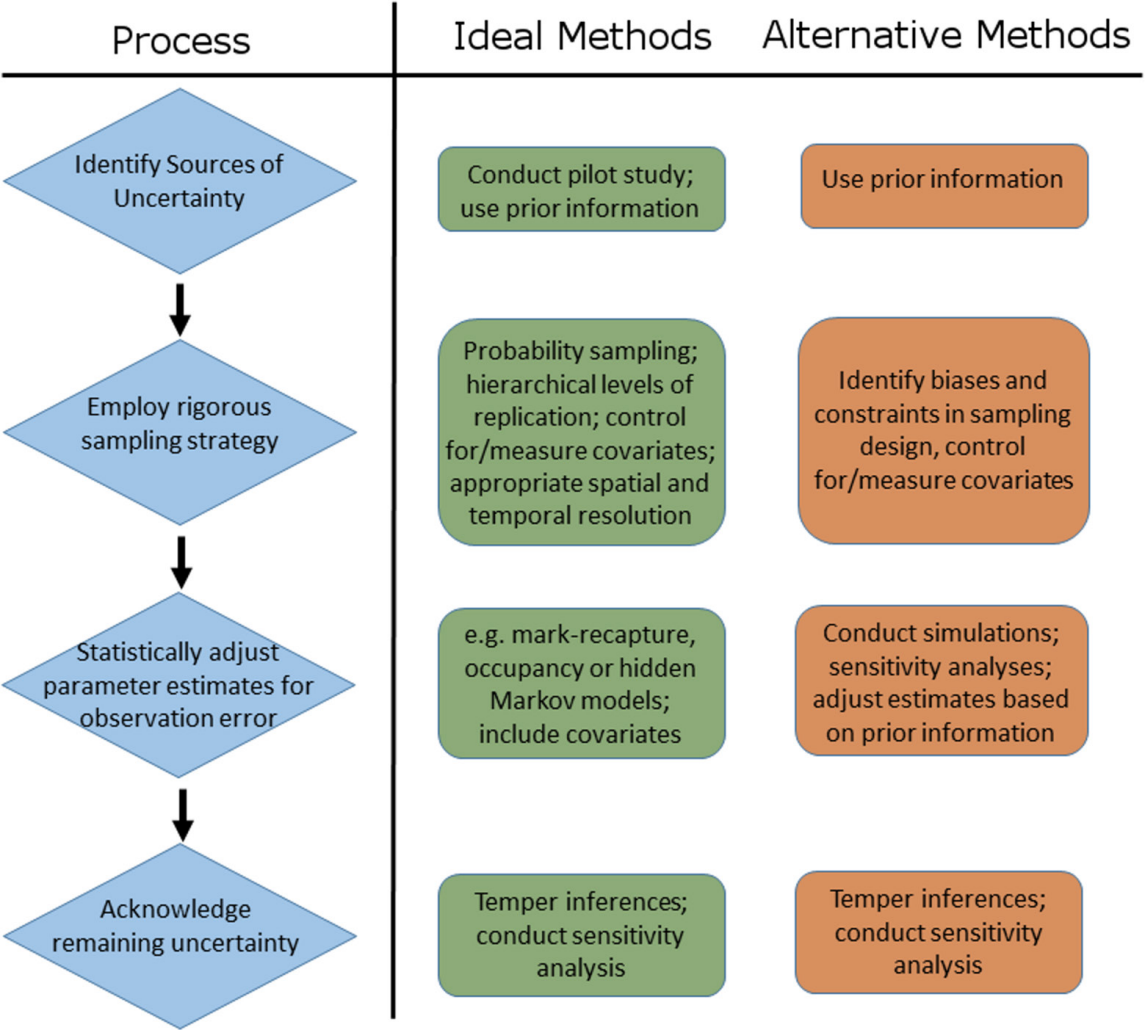
Schematic representation of the process of accounting for sources of uncertainty in disease ecology studies, including examples of ideal and alternative methods that can be used to address each step of the process.

Our review demonstrates that uncertainty in disease ecology studies arises because of the sampling and diagnostic procedures used and due to factors inherent to the biology and ecology of host-pathogen systems. Accordingly, the first step of any disease ecology study should be to identify potential sources of uncertainty and their likely magnitude (Figure 8). This will involve consulting prior information (previous studies, similar studies, historical literature) and, if resources permit, conducting a pilot study or power analysis to help determine optimal or efficient sampling strategies. Several aspects of a well-designed sampling strategy can *a priori* reduce the extent to which uncertainty plagues parameter estimates (72, 73). For example, minimising stochastic variation among samples (e.g., by ensuring that sampled individuals are representative of the population), controlling for known covariates of detection probability (26, 52, 74, 75), verifying the taxonomic resolution of the system, and evaluating the potential influence of non-detection of rare, hard to detect or cryptic species.

Subsequently, researchers should employ a sampling strategy that enables the application of statistical tools that can help adjust parameter estimates to account for uncertainty arising via imperfect detection (including incomplete sampling) and state misclassification (11, 15, 76; Figure 8). The statistical tools most commonly used fall under two broad frameworks. Occupancy models use repeated spatio-temporal sampling to estimate detection probabilities at multiple hierarchical levels and are a flexible means of obtaining estimates of disease parameters adjusted for multiple levels of uncertainty (11, 12, 15). For example, DiRenzo et al., (77) developed a novel hierarchical occupancy model to obtain estimates of the prevalence and infection intensity of Bd in a community of frogs, adjusting for detection errors arising both from field sampling and subsequent diagnostic testing procedures. Hidden Markov models (also termed multi-event mark-recapture models), meanwhile, model both individual detection probabilities and uncertainty in state assignment (16, 78), and are a powerful means of linking disease dynamics (estimates of transmission and recovery rates) to impacts on hosts and populations [via estimates of vital rates; (21, 79)]. Moreover, multi-event models can also provide a robust framework for improving diagnostic accuracy when diagnosis is imperfect, by enabling a probability-based, rather than a binary, classification of infection status. For example, Buzdugan et al., (51) used multi-event models to integrate multiple diagnostic test data alongside ecological and epidemiological information, while accounting for multiple sources of uncertainty (imperfect detection and false positive and false negative diagnoses) to generate an infection probability value for each individual in their study.

For studies that evaluate hosts or pathogen communities, existing tools stemming from biodiversity studies, such as non-parametric richness estimators, can be readily applied to estimate or account for the non-detection of species within species assemblages (80). Several previous studies provide comprehensive guidance to the design and application of these models in disease ecology and other studies (11, 15, 52, 75, 80–82), and we refer the interested reader to the more detailed discussions therein to develop further applications in disease systems.

Despite being widely advocated and employed in disease ecology studies, the application of analytical tools to obtain unbiased and more precise parameter estimates may not always be possible. Multi-event models require long-term, and often large, datasets on marked individuals (21, 51, 83), while occupancy models demand repeated sampling at every level of inference: from conducting multiple surveys at monitoring sites, to obtaining replicate tissue samples from sampled hosts, to performing multiple diagnostic assays of those replicate samples (11, 84). Numerous financial, logistic and even biological constraints can prevent such rigorous hierarchical sampling from being undertaken. Paradoxically, these methods also require reasonable detection probabilities (of hosts and pathogens) to estimate parameters of interest (11, 78). Logistic or financial constraints that limit the scale or frequency of sampling or the type or quality of data collected, or the presence of rare species, will therefore preclude the use of statistical adjustment to account for uncertainty in many cases. When such constraints preclude the use of such models to adjust parameter estimates for heterogeneous detectability or state misclassification, it may be possible to use prior information on detectability or state uncertainty from other studies or similar systems to adjust parameter estimates via Bayesian methods, or to use simulations and sensitivity analyses to assess the influence of a range of detectabilities or misclassification bias on parameter estimates and inferences (74, 75; Figure 8).

Ultimately, statistical tools can only adjust for uncertainty due to measured and identified sources of observation error (i.e., imperfect detection and state misclassification). A post-hoc statistical adjustment of infection rates will remain biased when infection is caused by multiple unidentified pathogen species, if the time span between sampling periods is greater than the average infection time for hosts, or if important species interactions within a multi-host disease system are overlooked. Thus, to truly account for multiple sources of uncertainty in disease ecology studies researchers must (i) have an intimate knowledge of the host-pathogen dynamics, the aetiology of the disease and the ecology of the system, (ii) employ a rigorous, biologically sound, replicated survey design, (iii) statistically adjust parameter estimates for known sources of uncertainty (or assess its influence on parameters otherwise), and (iv) acknowledge remaining sources of uncertainty (Figure 8). Where possible, the influence of remaining potential sources of uncertainty should be evaluated via simulation or sensitivity analyses (e.g., as discussed above), and if necessary, inferences on disease dynamics, impacts, distributions, and trends should be tempered accordingly (Figure 8). Somewhat ironically, the four steps listed above might be exactly what a study is trying to ascertain in the first place, paving the way for studies and methods that scale with, or incrementally improve, knowledge of a system utilising an adaptive or iterative approach. As such, depending on the state of knowledge of a particular system, each of the steps in this hierarchy of actions (Figure 8) represent potential future directions or avenues of enquiry for the system at hand.

## Conclusion

Reliable, unbiased and precise estimates of disease-relevant parameters are critical for disease monitoring and risk analysis; for predicting disease spread and dynamics; for understanding the ecological and evolutionary implications of pathogens in host populations; and for ensuring the success of conservation interventions and management actions (11, 15, 20, 85; Table 1). Over the last decade, disease ecologists have begun to acknowledge the importance of accounting for uncertainty when making inferences on natural disease systems (9, 11, 12, 14, 15, 20, 26, 52, 53, 76). To date, however, uncertainty in disease ecology studies has been considered primarily in terms of imperfect detection (of hosts or pathogens) or disease-state misclassification. In this review, we show that uncertainty in disease ecology studies extends beyond these components of observation error and can arise from multiple varied processes that pertain to aspects of the disease system, the study design, the methods used to study the system, and the state of knowledge of the system. Some of these processes, such as unidentified crypticity among vectors, hosts or pathogens, or a mismatch of sampling scales, may not be immediately apparent, and may not be adequately accounted for via statistical adjustments (11, 14). In this review, we have discussed the processes by which these varied sources of uncertainty can reduce the precision of, and introduce bias in, estimates of disease-relevant parameters. Importantly, we show that uncertainties in parameter estimates generated via one process may propagate through to others because of interactions between the numerous biological, methodological and environmental factors at play. Understanding how these interactions among sources of uncertainty affect the degree and direction of bias in disease-relevant parameters is a key challenge for this field, and we present a hierarchy of needs that could be tailored to individual study contexts in order to reveal next steps and future directions towards improving estimates of disease-relevant parameters.

Given the diverse set of factors that can contribute to uncertainty in disease ecology studies (Table 2), the extent of ecological variation in host-pathogen systems (e.g., Table 1), and the possibility of interactions among elements, assessments of the degree of potential bias and lack of precision in disease-relevant parameter estimates must be undertaken on a system-specific level. Nevertheless, some general guidelines are possible. The degree of uncertainty in disease ecology studies will be higher when the biology, ecology and dynamics of the system are complex or unresolved, when sampling effort is low, when sampling strategies are poorly designed (e.g., spatially or temporally biased) or undertaken at inappropriate spatial or temporal scales relative to disease dynamics, when diagnostic tools suffer from low or varying sensitivity or specificity, and when environmental covariates are complex or poorly resolved. Moreover, studies of endemic, invasive, or novel diseases may be at higher risk of uncertainty because the detectability of pathogens is often lower where disease prevalence and infection intensity are low or patchily distributed such as at invasion fronts.

Uncertainty in disease ecology studies is a certainty. In this review, we have identified a myriad of ways in which uncertainty can manifest when attempting to monitor pathogens and characterise disease dynamics in natural populations and have discussed appropriate sampling and analytical methods to account for or minimise their influence on estimates of disease-relevant parameters and identify future research priorities. We acknowledge that our list is not exhaustive, and that studies, particularly in novel systems, or that apply novel methodologies and technologies, will continue to encounter additional considerations. Nevertheless, this review should assist researchers and practitioners to navigate the pitfalls of uncertainty and strive towards more robust parameter estimates from which to make sound inferences and predictions in disease ecology.

## Author Contributions

SL and KM conceived of the design and ideas in this manuscript. SL performed the literature review. SL and KM wrote the manuscript.

## Conflict of Interest Statement

The authors declare that the research was conducted in the absence of any commercial or financial relationships that could be construed as a potential conflict of interest.

## Glossary of terms

Accuracy: the degree of closeness of a measurement of a quantity to its true value. It is a description of both systematic and random errors, such that high accuracy requires both high precision and low bias.
Bias: the tendency of measurements of a quantity to shift systematically in one direction from the true value. It is a description of systematic errors.
Detectability: probability that an organism will be encountered (captured or seen) in its natural habitat during a survey.
Disease-induced impacts: changes in host fitness, behaviour, or physiological performance caused by pathogen infection.
Disease state uncertainty: occurs when disease state assignment is subject to error (disease state misclassification) or not known for a portion of the sampled individuals.
Force of infection: the instantaneous rate at which susceptible individuals acquire infection.
Heterogeneity in detectability: occurs when there is variation in the probability of detecting organisms in their natural habitat.
Imperfect detection: occurs when the probability of detecting an organism in its natural habitat is less than one.
Incidence: the rate at which new infections occur in a population during a specified period.
Macroparasite: a pathogen that does not multiply within its host (e.g., helminths).
Microparasite: a pathogen that does multiply within its host (e.g., viruses).
Pathogen load: is a measure of the number of parasites that a host organism harbours. Also referred to as the intensity of infection.
Persistence time: length of time an infectious agent persists in the environment.
Precision: the degree of agreement in a series of measurements of a quantity. It is a description of random errors; a measure of statistical variability.
Prevalence: the proportion of a population that are infected at a point in time.
Risk of infection: the probability of becoming infected given that exposure to an infectious pathogen has occurred.
Sensitivity: the ability of a test to identify correctly those with the disease (true positive rate).
Specificity: the ability of the test to identify correctly those without the disease (true negative rate).
Taxonomic crypticity: occurs when individuals are phenotypically indistinguishable but genetically distinct.
Transmission rate: the rate at which pathogens pass from infected hosts to other individuals in a population, or the rate at which hosts contact infectious agents in the environment, during a specified period.
Uncertainty: occurs when incomplete knowledge of a system, or irreducible naturally occurring noise, prevents ascertainment of the truth. Uncertainty leads to a lack of precision and bias in measurements obtained.
