# Supplementary material for: The Certainty of Uncertainty: Potential Sources of Bias and Imprecision in Disease Ecology Studies
Source: Front Vet Sci. 2018 May 22;5:90. doi: 10.3389/fvets.2018.00090 (PMC5972326; doi:10.3389/fvets.2018.00090)
Supplement: Supplementary file 1 [file DataSheet1.docx]

**Appendix 1**

We conducted a literature survey using the Web of Science for articles published from 1945 to 2017 and limited to the research area ecology (i.e. SU=ecology) with the following search terms in the title, abstract, or keywords: [“pathogen*” OR “disease*” OR infect* OR parasit*] AND [“non-detection" OR "detection prob*" OR "heterogeneity in detection" OR "imperfect detection" OR "uncertainty" OR "state misclassification"] AND [wild OR wildlife OR natural], which yielded 167 articles. From these articles, and additional relevant articles referenced within them, we identified the most common sources of potential uncertainty in estimates of disease-relevant parameters encountered or discussed in the literature. Below we list the articles we used to categorise source of uncertainty in disease ecology studies.

**Results of literature search**

Abad-Franch, F., Valenca-Barbosa, C., Sarquis, O., and Lima, M.M. (2014). All That glisters is not gold: sampling-process uncertainty in disease-vector surveys with false negative and false positive detections. *Plos Neglected Tropical Diseases* 8(9)**,** 11. doi: 10.1371/journal.pntd.0003187.

Adams, M.J., Chelgren, N.D., Reinitz, D., Cole, R.A., Rachowicz, L.J., Galvan, S., et al. (2010). Using occupancy models to understand the distribution of an amphibian pathogen, Batrachochytrium dendrobatidis. *Ecological Applications* 20(1)**,** 289-302. doi: 10.1890/08-2319.1.

Altizer, S., Dobson, A., Hosseini, P., Hudson, P., Pascual, M., and Rohani, P. (2006). Seasonality and the dynamics of infectious diseases. *Ecology Letters* 9(4)**,** 467-484. doi: 10.1111/j.1461-0248.2005.00879.x.

Anderson, R.M. and May, R.M. (1978). Regulation and Stability of Host-Parasite Population Interactions: I. Regulatory Processes. *Journal of Animal Ecology* 47(1), 219-247. doi: 10.2307/3933.

Anthony, S.J., Epstein, J.H., Murray, K.A., Navarrete-Macias, I., Zambrana-Torrelio, C.M., Solovyov, A., et al. (2013). A strategy to estimate unknown viral diversity in mammals. *mBio* 4(5). doi: 10.1128/mBio.00598-13.

Archaux, F., Henry, P.Y., and Gimenez, O. (2012). When can we ignore the problem of imperfect detection in comparative studies? *Methods in Ecology and Evolution* 3(1)**,** 188-194. doi: 10.1111/j.2041-210X.2011.00142.x.

Arthur, A; Ramsey, D; Efford, M (2004). Impact of bovine tuberculosis on a population of brushtail possums (*Trichosurus vulpecula Kerr*) in the Orongorongo Valley, New Zealand. *Wildlife Research* 31(4), 389-395. doi: 10.1071/WR03097.

Backlin, Adam R.; Hitchcock, Cynthia J.; Gallegos, Elizabeth A.; Yee, Julie L.; Fisher, Robert N. (2015). The precarious persistence of the Endangered Sierra Madre yellow-legged frog *Rana muscosa* in southern California, USA. *Oryx* 49(1), 157-164. doi:10.1017/S003060531300029X.

Banks-Leite, C., Pardini, R., Boscolo, D., Cassano, C.R., Puttker, T., Barros, C.S., et al. (2014). Assessing the utility of statistical adjustments for imperfect detection in tropical conservation science. *Journal of Applied Ecology* 51(4)**,** 849-859. doi: 10.1111/1365-2664.12272.

Barber-Meyer, Shannon M. (2010). Dealing with the clandestine nature of wildlife-trade market surveys. Conservation Biology 24(4), 918-923. doi: 10.1111/j.1523-1739.2010.01500.x.

Beasley, James C.; Beatty, William S.; Atwood, Todd C.; Johnson, Shylo R.; Rhodes, Olin E., Jr. (2012). A comparison of methods for estimating raccoon abundance: Implications for disease vaccination programs. Journal of Wildlife Management 76(6), 1290-1297. doi: 10.1002/jwmg.379.

Bass, D., Stentiford, G.D., Littlewood, D.T.J., and Hartikainen, H. (2015). Diverse applications of environmental DNA methods in parasitology. *Trends in Parasitology* 31(10)**,** 499-513. doi: 10.1016/j.pt.2015.06.013.

Beldomenico, P.M., and Begon, M. (2010). Disease spread, susceptibility and infection intensity: vicious circles? *Trends in Ecology & Evolution* 25(1)**,** 21-27. doi: 10.1016/j.tree.2009.06.015.

Benavides, J.A., Caillaud, D., Scurlock, B.M., Maichak, E.J., Edwards, W.H., and Cross, P.C. (2017). Estimating loss of *Brucella abortus* antibodies from age-specific serological data in elk. *Ecohealth* 14(2), 234-243. doi: 10.1007/s10393-017-1235-z.

Bollaerts, K., Aerts, M., Shkedy, Z., Faes, C., Van der Stede, Y., Beutels, P., et al. (2012). Estimating the population prevalence and force of infection directly from antibody titres. *Statistical Modelling* 12(5)**,** 441-462. doi: 10.1177/1471082x12457495.

Bradshaw, C. J., Miller, P. S., Lacy, R.C., Watts, M.J., Verant, M. L., Pollak, J. P., Fordham, D. A.; Prowse, T. A. A.; Brook, B. W. (2012) Novel coupling of individual-based epidemiological and demographic models predicts realistic dynamics of tuberculosis in alien buffalo. *Journal Of Applied Ecology* 49(1), 268-277. doi: 10.1111/j.1365-2664.2011.02081.x

Budischak, S.A., Jolles, A.E., and Ezenwa, V.O. (2012). Direct and indirect costs of co-infection in the wild: Linking gastrointestinal parasite communities, host hematology, and immune function. *International Journal for Parasitology: Parasites and Wildlife* 1(Supplement C)**,** 2-12. doi: 10.1016/j.ijppaw.2012.10.001.

Butler, C.J., Edwards, W.H., Jennings-Gaines, J.E., Killion, H.J., Wood, M.E., McWhirter, D.E., et al. (2017). Assessing respiratory pathogen communities in bighorn sheep populations: Sampling realities, challenges, and improvements. *Plos One* 12(7)**,** 21. doi: 10.1371/journal.pone.0180689.

Buzdugan, S.N., Vergne, T., Grosbois, V., Delahay, R.J., and Drewe, J.A. (2017). Inference of the infection status of individuals using longitudinal testing data from cryptic populations: Towards a probabilistic approach to diagnosis. *Scientific Reports* 7(1). doi: 10.1038/s41598-017-00806-4.

Byrne, Andrew W.; Quinn, John L.; O'Keeffe, James J.; Green, Stuart; Sleeman, D. Paddy; Martin, S. Wayne; Davenport, John. (2014). Large-scale movements in European badgers: has the tail of the movement kernel been underestimated? *Journal Of Animal Ecology* 83(4), 991-1001. doi: 10.1111/1365-2656.12197.

Canessa, S., Martel, A., and Pasmans, F. (2014). Designing screening protocols for amphibian disease that account for imperfect and variable capture rates of individuals. *Ecological Applications* 24(5)**,** 1204-1212.

Cashins, S.D., Philips, A., Skerratt, L.F. (2015) Using site-occupancy models to prepare for the spread of chytridiomyosis and identify factors affecting detectability of a cryptic susceptible species, the Tasmanian tree frog. *Wildlife Research* 42(5), 405-413. doi: 10.1071/WR14183.

Chakraborty, S. (2013) Migrate or evolve: options for plant pathogens under climate change. *Global Change Biology* 19(7), 1985-2000. doi: 10.1111/gcb.12205.

Chambert, T. M., D. A. W.; Nichols, J. D. (2015). Modeling false positive detections in species occurrence data under different study designs. *Ecology* 96(2), 332-339. doi: 10.1890/14-1507.1.

Chambert, T. M., Staszewski, V., Lobato, E., Choquet, R., Carrie, C., McCoy, K.D., Tveraa, T., Boulinier, T. (2012) Exposure of black-legged kittiwakes to Lyme disease spirochetes: dynamics of the immune status of adult hosts and effects on their survival. *Journal Of Animal Ecology* 81(5), 986-995. doi: 10.1111/j.1365-2656.2012.01979.x.

Chambert, T., Staszewski, V., Lobato, E., Choquet, R., Carrie, C., McCoy, K.D., et al. (2012). Exposure of black-legged kittiwakes to Lyme disease spirochetes: dynamics of the immune status of adult hosts and effects on their survival. *Journal of Animal Ecology* 81(5)**,** 986-995. doi: 10.1111/j.1365-2656.2012.01979.x.

Chao, A., and Jost, L. (2015). Estimating diversity and entropy profiles via discovery rates of new species. *Methods in Ecology and Evolution* 6(8)**,** 873-882. doi: 10.1111/2041-210X.12349.

Chestnut, T. A., C.; Popa, R.; Blaustein, A. R.; Voytek, M.; Olson, D. H.; Kirshtein, J. (2014) Heterogeneous occupancy and density estimates of the pathogenic fungus *Batrachochytrium dendrobatidis* in waters of North America. *Plos One* 9(9), 11. doi: 10.1371/journal.pone.0106790.

Choquet, R., Rouan, L., and Pradel, R. (2009). Program E-SURGE: a software application for fitting multievent models. *Modeling Demographic Processes in Marked Populations* 3**,** 845-865. doi: 10.1007/978-0-387-78151-8_39.

Clark, N.J., Clegg, S.M. (2017) Integrating phylogenetic and ecological distances reveals new insights into parasite host specificity. *Molecular Ecology* 26(11), 3074-3086. doi: 10.1111/mec.14101.

Clement, M.J. (2016). Designing occupancy studies when false-positive detections occur. *Methods in Ecology and Evolution* 7(12)**,** 1538-1547. doi: 10.1111/2041-210x.12617.

Colvin, M.E., Peterson, J.T., Kent, M.L., and Schreck, C.B. (2015). Occupancy modeling for improved accuracy and understanding of pathogen prevalence and dynamics. *PLOS ONE* 10(3)**,** e0116605. doi: 10.1371/journal.pone.0116605.

Conn, P.B., and Cooch, E.G. (2009). Multistate capture-recapture analysis under imperfect state observation: an application to disease models. *Journal of Applied Ecology* 46(2)**,** 486-492. doi: 10.1111/j.1365-2664.2008.01597.x.

Conn, P. B. C., E. G.; Caley, P. (2012) Accounting for detection probability when estimating force-of-infection from animal encounter data. *Journal Of Ornithology* 152(), S511-S520 doi: 10.1007/s10336-010-0591-z.

Conte, A., Gilbert, M., Goffredo, M. (2009) Eight years of entomological surveillance in Italy show no evidence of *Culicoides imicola* geographical range expansion. *Journal Of Applied Ecology* 46(6), 1332-1339. doi: 10.1111/j.1365-2664.2009.01723.x.

Cooch, E.G., Conn, P.B., Ellner, S.P., Dobson, A.P., and Pollock, K.H. (2012). Disease dynamics in wild populations: modeling and estimation: a review. *Journal of Ornithology* 152(2)**,** 485-509. doi: 10.1007/s10336-010-0636-3.

Cornwell, E.R., Anderson, G.B., Coleman, D., Getchell, R.G., Groocock, G.H., Warg, J.V., Cruz, A.M., Casey, J.W., Bain, M.B., Bowser, P.R. (2015) Applying multi-scale occupancy models to infer host and site occupancy of an emerging viral fish pathogen in the Great Lakes. *Journal Of Great Lakes Research* 41(2), 520-529. doi: 10.1016/j.jglr.2015.01.002.

Correia-Gomes, C.E.T., Bailey, T., Brazdil, P., Alban, L., Niza-Ribeiro, J. (2014) Transmission parameters estimated for *Salmonella typhimurium* in swine using susceptible-infectious-resistant models and a Bayesian approach *Bmc Veterinary Research* 10(11). doi: 10.1186/1746-6148-10-101.

Crone, E.E.. Pickering, D., Schultz, C.B. (2007) Can captive rearing promote recovery of endangered butterflies? An assessment in the face of uncertainty. *Biological Conservation* 139(43132), 103-112. doi: 10.1016/j.biocon.2007.06.007.

Cross, P.C., Maichak, E.J., Rogerson, J.D., Irvine, K.M., Jones, J.D., Heisey, D.M., Edwards, W.H., Scurlock, B.M. (2015) Estimating the phenology of elk brucellosis transmission with hierarchical models of cause-specific and baseline hazards. *Journal Of Wildlife Management* 79(5), 739-748. doi: 10.1002/jwmg.883.

DiRenzo, G. V. C. G., Evan H.; Longo, Ana V.; Che-Castaldo, Christian; Zamudio, Kelly R.; Lips, Karen R. (2018) Imperfect pathogen detection from non-invasive skin swabs biases disease inference. *Methods In Ecology And Evolution* 9(2), 380-389. doi: 10.1111/2041-210X.12868.

Doan, T. N. K., D. C. M.; Marshall, C.; Kirkpatrick, C. M. J.; McBryde, E. S. (2015) Characterising the Transmission Dynamics of Acinetobacter baumannii in Intensive Care Units Using Hidden Markov Models. *Plos One* 10(7), 15. doi: 10.1371/journal.pone.0132037.

Eads, D.A., Biggins, D.E., Antolin, M.F., Long, D.H., Huyvaert, K.P., and Gage, K.L. (2015). Prevalence of the generalist flea *Pulex simulans* on black-tailed prairie dogs (*Cynomys ludovicianus*) in New Mexico, USA: the importance of considering imperfect detection. *Journal of Wildlife Diseases* 51(2)**,** 498-502. doi: 10.7589/2014-07-178.

Ebert, Cornelia; Knauer, Felix; Spielberger, Bettina; Thiele, Bernhard; Hohmann, Ulf. (2012). Estimating wild boar *Sus scrofa* population size using faecal DNA and capture-recapture modelling. *Wildlife Biology* 18(2), 142-152 doi: 10.2981/11-002.

Drewe, J.A. (2009). Who infects whom? Social networks and tuberculosis transmission in wild meerkats. *Proceedings of the Royal Society B: Biological Sciences*. doi: 10.1098/rspb.2009.1775.

Elmore, S.A., Huyvaert, K.P., Bailey, L.L., Iqbal, A., Su, C., Dixon, B.R., et al. (2016). Multi-scale occupancy approach to estimate Toxoplasma gondii prevalence and detection probability in tissues: an application and guide for field sampling. *International Journal for Parasitology* 46(9)**,** 563-570. doi: 10.1016/j.ijpara.2016.04.003.

Faustino, C.R., Jennelle, C.S., Connolly, V., Davis, A.K., Swarthout, E.C., Dhondt, A.A., et al. (2004). Mycoplasma gallisepticum infection dynamics in a house finch population: seasonal variation in survival, encounter and transmission rate. *Journal of Animal Ecology* 73(4)**,** 651-669. doi: 10.1111/j.0021-8790.2004.00840.x.

George, D.B., Webb, C.T., Farnsworth, M.L., O'Shea, T.J., Bowen, R.A., Smith, D.L., et al. (2011). Host and viral ecology determine bat rabies seasonality and maintenance. *Proceedings of the National Academy of Sciences* 108(25)**,** 10208-10213. doi: 10.1073/pnas.1010875108.

Gibb, R., Moses, L.M., Redding, D.W., and Jones, K.E. (2017). Understanding the cryptic nature of Lassa fever in West Africa. *Pathogens and Global Health* 111(6)**,** 276-288. doi: 10.1080/20477724.2017.1369643.

Gilbert, A.T., Fooks, A.R., Hayman, D.T.S., Horton, D.L., Muller, T., Plowright, R., et al. (2013). Deciphering Serology to Understand the Ecology of Infectious Diseases in Wildlife. *Ecohealth* 10(3)**,** 298-313. doi: 10.1007/s10393-013-0856-0.

Gomez-Diaz, E., Doherty, P.F., Duneau, D., and McCoy, K.D. (2010). Cryptic vector divergence masks vector-specific patterns of infection: an example from the marine cycle of Lyme borreliosis. *Evolutionary Applications* 3(4)**,** 391-401. doi: 10.1111/j.1752-4571.2010.00127.x.

Grogan, L.F., Phillott, A.D., Scheele, B.C., Berger, L., Cashins, S.D., Bell, S.C., et al. (2016). Endemicity of chytridiomycosis features pathogen overdispersion. *Journal of Animal Ecology* 85(3)**,** 806-816. doi: 10.1111/1365-2656.12500.

Gurtler, R.E., and Cardinal, M.V. (2015). Reservoir host competence and the role of domestic and commensal hosts in the transmission of Trypanosoma cruzi. *Acta Trop* 151**,** 32-50. doi: 10.1016/j.actatropica.2015.05.029.

Haapkyla, J., Unsworth, R.K.F., Flavell, M., Bourne, D.G., Schaffelke, B., and Willis, B.L. (2011). Seasonal rainfall and runoff promote coral disease on an inshore reef. *Plos One* 6(2)**,** 10. doi: 10.1371/journal.pone.0016893.

Heisey, D.M., Jennelle, C.S., Russell, R.E., and Walsh, D.P. (2014). Using Auxiliary Information to Improve Wildlife Disease Surveillance When Infected Animals Are Not Detected: A Bayesian Approach. *PLOS ONE* 9(3)**,** e89843. doi: 10.1371/journal.pone.0089843.

Hosseini, P.R., Dhondt, A.A., and Dobson, A. (2004). Seasonality and wildlife disease: how seasonal birth, aggregation and variation in immunity affect the dynamics of <em>Mycoplasma gallisepticum</em> in house finches. *Proceedings of the Royal Society of London. Series B: Biological Sciences* 271(1557)**,** 2569-2577. doi: 10.1098/rspb.2004.2938.

Ishtiaq, F., Rao, M., Huang, X., and Bensch, S. (2017). Estimating prevalence of avian haemosporidians in natural populations: a comparative study on screening protocols. *Parasites & Vectors* 10**,** 10. doi: 10.1186/s13071-017-2066-z.

Jennelle, C.S., Cooch, E.G., Conroy, M.J., and Senar, J.C. (2007). State-specific detection probabilities and disease prevalence. *Ecological Applications* 17(1)**,** 154-167. doi: 10.1890/1051-0761(2007)017[0154:sdpadp]2.0.co;2.

Johnson, B.J., Munafo, K., Shappell, L., Tsipoura, N., Robson, M., Ehrenfeld, J., et al. (2012). The roles of mosquito and bird communities on the prevalence of West Nile virus in urban wetland and residential habitats. *Urban Ecosyst* 15(3)**,** 513-531. doi: 10.1007/s11252-012-0248-1.

Johnson, P.T.J., Ostfeld, R.S., and Keesing, F. (2015). Frontiers in research on biodiversity and disease. *Ecology Letters* 18(10)**,** 1119-1133. doi: 10.1111/ele.12479.

Jolles, A.E., Ezenwa, V.O., Etienne, R.S., Turner, W.C., and Olff, H. (2008). Interactions between macroparasites and microparasites drive infection patterns in free-ranging African buffalo. *Ecology* 89(8)**,** 2239-2250. doi: 10.1890/07-0995.1.

Kilpatrick, A.M., Daszak, P., Jones, M.J., Marra, P.P., and Kramer, L.D. (2006). Host heterogeneity dominates West Nile virus transmission. *Proc Biol Sci* 273(1599)**,** 2327-2333. doi: 10.1098/rspb.2006.3575.

Kollias, G.V., Sydenstricker, K.V., Kollias, H.W., Ley, D.H., Hosseini, P.R., Connolly, V., et al. (2004). Experimental infection of house finches with *Mycoplasma gallisepticum*. *Journal of Wildlife Diseases* 40(1)**,** 79-86.

Lachish, S., Gopalaswamy, A.M., Knowles, S.C.L., and Sheldon, B.C. (2012). Site-occupancy modelling as a novel framework for assessing test sensitivity and estimating wildlife disease prevalence from imperfect diagnostic tests. *Methods in Ecology and Evolution* 3(2)**,** 339-348. doi: 10.1111/j.2041-210X.2011.00156.x.

Lachish, S., Jones, M., and McCallum, H. (2007). The impact of disease on the survival and population growth rate of the Tasmanian devil. *Journal of Animal Ecology* 76(5)**,** 926-936. doi: 10.1111/j.1365-2656.2007.01272.x.

Lachish, S., Knowles, S.C.L., Alves, R., Wood, M.J., and Sheldon, B.C. (2011a). Fitness effects of endemic malaria infections in a wild bird population: the importance of ecological structure. *Journal of Animal Ecology* 80(6)**,** 1196-1206. doi: 10.1111/j.1365-2656.2011.01836.x.

Lachish, S., Knowles, S.C.L., Alves, R., Wood, M.J., and Sheldon, B.C. (2011b). Infection dynamics of endemic malaria in a wild bird population: parasite species-dependent drivers of spatial and temporal variation in transmission rates. *Journal of Animal Ecology* 80(6)**,** 1207-1216. doi: 10.1111/j.1365-2656.2011.01893.x.

Langwig, K.E., Frick, W.F., Reynolds, R., Parise, K.L., Drees, K.P., Hoyt, J.R., et al. (2015). Host and pathogen ecology drive the seasonal dynamics of a fungal disease, white-nose syndrome. *Proceedings of the Royal Society B: Biological Sciences* 282(1799)**,** 20142335. doi: 10.1098/rspb.2014.2335.

Levi, T., Keesing, F., Holt, R.D., Barfield, M., and Ostfeld, R.S. (2016). Quantifying dilution and amplification in a community of hosts for tick-borne pathogens. *Ecol Appl* 26(2)**,** 484-498.

Loss, S.R., Hamer, G.L., Walker, E.D., Ruiz, M.O., Goldberg, T.L., Kitron, U.D., et al. (2009). Avian host community structure and prevalence of West Nile virus in Chicago, Illinois. *Oecologia* 159(2)**,** 415-424. doi: 10.1007/s00442-008-1224-6.

Madelaire, C.B., José da Silva, R., and Ribeiro Gomes, F. (2013). Calling behavior and parasite intensity in treefrogs, *Hypsiboas prasinus*. *Journal of Herpetology* 47(3)**,** 450-455. doi: 10.1670/11-315.

Marescot, L., Benhaiem, S, Gimenez, O., Hofer, H., Lebreton, J-D, Olarte-Castillo, X.A., Kramer-Schadt, S., East, M.L. (2018). Social status mediates the fitness costs of infection with canine distemper virus in a social carnivore. *Functional Ecology*. doi: 10.1111/1365-2435.13059.

McClintock, B.T., Nichols, J.D., Bailey, L.L., MacKenzie, D.I., Kendall, W.L., and Franklin, A.B. (2010). Seeking a second opinion: uncertainty in disease ecology. *Ecology Letters* 13(6)**,** 659-674. doi: 10.1111/j.1461-0248.2010.01472.x.

Miller, D.A., Nichols, J.D., McClintock, B.T., Grant, E.H.C., Bailey, L.L., and Weir, L.A. (2011). Improving occupancy estimation when two types of observational error occur: non-detection and species misidentification. *Ecology* 92(7)**,** 1422-1428. doi: 10.1890/10-1396.1.

Miller, D.A.W., Bailey, L.L., Grant, E.H.C., McClintock, B.T., Weir, L.A., and Simons, T.R. (2015). Performance of species occurrence estimators when basic assumptions are not met: a test using field data where true occupancy status is known. *Methods in Ecology and Evolution* 6(5)**,** 557-565. doi: 10.1111/2041-210x.12342.

Miller, D.A.W., Talley, B.L., Lips, K.R., and Grant, E.H. (2012). Estimating patterns and drivers of infection prevalence and intensity when detection is imperfect and sampling error occurs. *Methods in Ecology and Evolution* 3(5)**,** 850-859. doi: 10.1111/j.2041-210X.2012.00216.x.

Murray, K.A., Retallick, R.W.R., Puschendorf, R., Skerratt, L.F., Rosauer, D., McCallum, H.I., et al. (2011). Assessing spatial patterns of disease risk to biodiversity: implications for the management of the amphibian pathogen, Batrachochytrium dendrobatidis. *Journal of Applied Ecology* 48(1)**,** 163-173. doi: 10.1111/j.1365-2664.2010.01890.x.

Murray, K.A., Skerratt, L.F., Speare, R., and McCallum, H. (2009). Impact and dynamics of disease in species threatened by the amphibian chytrid fungus, *Batrachochytrium dendrobatidis*. *Conservation Biology* 23(5)**,** 1242-1252. doi: 10.1111/j.1523-1739.2009.01211.x.

Nichols, J. D., Hollmen, T.E., Grand, J.B. (2017). Monitoring for the management of disease risk in animal translocation programmes.*EcoHealth* 14(1), 156-166. doi: 10.1007/s10393-015-1094-4.

Nusser, S.M., Clark, W.R., Otis, D.L., and Huang, L. (2008). Sampling considerations for disease surveillance in wildlife populations. *The Journal of Wildlife Management* 72(1)**,** 52-60. doi: 10.2193/2007-317.

Orynbayev, M.B., Beauvais, W., Sansyzbay, A.R., Rystaeva, R.A., Sultankulova, K.T., Kerimbaev, A.A., et al. (2016). Seroprevalence of infectious diseases in saiga antelope (Saiga tatarica tatarica) in Kazakhstan 2012-2014. *Prev Vet Med* 127**,** 100-104. doi: 10.1016/j.prevetmed.2016.03.016.

Palinauskas, V., Žiegytė, R., Ilgūnas, M., Iezhova, T.A., Bernotienė, R., Bolshakov, C., et al. (2015). Description of the first cryptic avian malaria parasite, Plasmodium homocircumflexum n. sp., with experimental data on its virulence and development in avian hosts and mosquitoes. *International Journal for Parasitology* 45(1)**,** 51-62. doi: 10.1016/j.ijpara.2014.08.012.

Phillips, S.J., Dudik, M., Elith, J., Graham, C.H., Lehmann, A., Leathwick, J., et al. (2009). Sample selection bias and presence-only distribution models: implications for background and pseudo-absence data. *Ecological Applications* 19(1)**,** 181-197.

Plowright, R.K., Manlove, K.R., Besser, T.E., Paez, D.J., Andrews, K.R., Matthews, P.E., et al. (2017). Age-specific infectious period shapes dynamics of pneumonia in bighorn sheep. *Ecology Letters* 20(10)**,** 1325-1336. doi: 10.1111/ele.12829.

Puschendorf, R., J Hoskin, C., Cashins, S., McDonald, K., Skerratt, L., Vanderwal, J., et al. (2011). *Environmental refuge from disease-driven amphibian extinction.*

Reye, A.L., Hubschen, J.M., Sausy, A., and Muller, C.P. (2010). Prevalence and seasonality of tick-borne pathogens in questing *Ixodes ricinus* ticks from Luxembourg. *Applied and Environmental Microbiology* 76(9)**,** 2923-2931. doi: 10.1128/aem.03061-09.

Ruiz-Gutierrez, V., Hooten, M.B., and Grant, E.H.C. (2016). Uncertainty in biological monitoring: a framework for data collection and analysis to account for multiple sources of sampling bias. *Methods in Ecology and Evolution* 7(8)**,** 900-909. doi: 10.1111/2041-210x.12542.

Samuel, M.D., and Storm, D.J. (2016). Chronic wasting disease in white-tailed deer: infection, mortality, and implications for heterogeneous transmission. *Ecology* 97(11)**,** 3195-3205. doi: 10.1002/ecy.1538.

Senar, J.C. (2004). Multi-state analysis of the impacts of avian pox on a population of Serins (Serinus serinus): the importance of estimating recapture rates. *Animal Biodiversity and Conservation* 27.1**,** 133-146.

Shin, J., Bataille, A., Kosch, T.A., and Waldman, B. (2014). Swabbing often fails to detect amphibian Chytridiomycosis under conditions of low infection load. *PLoS One* 9(10)**,** e111091. doi: 10.1371/journal.pone.0111091.

Sonia Altizer, Charles L. Nunn, Peter H. Thrall, John L. Gittleman, Janis Antonovics, Andrew A. Cunningham, et al. (2003). Social Organization and Parasite Risk in Mammals: Integrating Theory and Empirical Studies. *Annual Review of Ecology, Evolution, and Systematics* 34(1)**,** 517-547. doi: 10.1146/annurev.ecolsys.34.030102.151725.

Susi, H., Barrès, B., Vale, P.F., and Laine, A.-L. (2015). Co-infection alters population dynamics of infectious disease. *Nature Communications* 6**,** 5975. doi: 10.1038/ncomms6975.

Svensson-Coelho, M., Silva, G.T., Santos, S.S., Miranda, L.S., Araujo-Silva, L.E., Ricklefs, R.E., et al. (2016). Lower detection probability of avian Plasmodium in blood compared to other tissues. *Journal of Parasitology* 102(5)**,** 559-561. doi: 10.1645/16-8.

Valenca-Barbosa, C., Lima, M.M., Sarquis, O., Bezerra, C.M., and Abad-Franch, F. (2014). Modeling disease vector occurrence when detection is imperfect II: drivers of site-occupancy by synanthropic *Triatoma brasiliensis* in the Brazilian northeast. *Plos Neglected Tropical Diseases* 8(5)**,** 12. doi: 10.1371/journal.pntd.0002861.

Voordouw, M.J., Lachish, S., and Dolan, M.C. (2015). The lyme disease pathogen has no effect on the survival of its rodent reservoir host. *Plos One* 10(2). doi: 10.1371/journal.pone.0118265.

Weber, N., Carter, S.P., Dall, S.R.X., Delahay, R.J., McDonald, J.L., Bearhop, S., et al. (2013). Badger social networks correlate with tuberculosis infection. *Current Biology* 23(20)**,** R915-R916. doi: 10.1016/j.cub.2013.09.011.

Wilson, K., Grenfell, B.T., and Shaw, D.J. (1996). Analysis of aggregated parasite distributions: A comparison of methods. *Functional Ecology* 10(5)**,** 592-601. doi: 10.2307/2390169.

Wood, C.L., and Lafferty, K.D. (2013). Biodiversity and disease: a synthesis of ecological perspectives on Lyme disease transmission. *Trends Ecol Evol* 28(4)**,** 239-247. doi: 10.1016/j.tree.2012.10.011.

Zipkin, E.F., Jennelle, C.S., and Cooch, E.G. (2010). A primer on the application of Markov chains to the study of wildlife disease dynamics. *Methods in Ecology and Evolution* 1(2)**,** 192-198. doi: 10.1111/j.2041-210X.2010.00018.x.
